# The role of hypoxia in chronic kidney disease: a nuanced perspective

**DOI:** 10.1097/MNH.0000000000000989

**Published:** 2024-04-10

**Authors:** Anna Faivre, Sophie de Seigneux

**Affiliations:** a Service de néphrologie, Département des Spécialités de Médecine Interne, Hôpitaux Universitaires de Genève; b Département de Physiologie Cellulaire et Métabolisme, Université de Genève, Genève, Suisse

**Keywords:** chronic kidney disease, hypoxia-inducible factor, hypoxia, prolyl-4-hydroxylase domain inhibitors, SGLT2 inhibitors

## Abstract

**Purpose of review:**

This review critically examines the role of hypoxia in chronic kidney disease (CKD). While traditionally viewed as detrimental, recent insights suggest a more nuanced understanding of hypoxia's role during renal disease.

**Recent findings:**

Emerging evidence challenges the traditional view that hypoxia is universally harmful in CKD context. We review here the recent evidence about hypoxia and HIF activation in CKD. We also discuss the effect of hypoxia on the renal tissue, and the relative inhibition of different HIF isoforms. Recent advancements in therapies, such as HIF prolyl hydroxylase inhibitors (HIF-PHIs) and sodium-glucose cotransporter 2 (SGLT2) inhibitors seem to target the HIF pathway. These drugs impact anemia associated with CKD

but also renoprotection, hinting at a more complex interplay between hypoxia, HIF activation, and renal health.

**Summary:**

A certain level of hypoxia and specific HIF pathway activation, especially HIF-α, can be beneficial in CKD progression. Therapeutic strategies targeting HIF stabilization, such as with HIF-PHIs and SGLT2 inhibitors, offer promising avenues for enhancing renal protection. Future investigations should aim at better understanding the precise effects on HIF pathway and optimize their clinical application to improve outcomes for CKD patients.

## INTRODUCTION

Chronic kidney disease (CKD) is a widespread health issue globally, contributing significantly to morbidity, mortality, and healthcare expenditure. The mechanisms driving CKD progression postinjury, particularly the involvement of hypoxia and hypoxia-inducible factor (HIF) transcription factors, have been extensively discussed. The initial hypothesis by Fine was that hypoxia triggers a harmful cycle of CKD progression by inducing inflammation and fibrosis related to uncontrolled HIF activation. This hypothesis was primarily rooted in the observed decline of peritubular capillaries, altering perfusion of the kidney tissue [1]. Nevertheless, several key energy consuming functions of tubular cells, such as sodium transport, are altered during CKD, likely decreasing oxygen consumption by this compartment. In addition, there are evidence showing that the HIF pathway is not activated during chronic kidney disease, either because of abnormal hypoxia sensing, or due to other inhibitory factors. This is in line with EPO stimulation by HIF stabilizers or SGLT2 inhibitors.

We discuss in this review these controversial data about the presence of hypoxia during CKD, its pathogenic role, and the state of HIF pathway activation. We then discuss the therapeutic options to modulate HIF during CKD, and especially HIF stabilizers and SGLT2 inhibitors. 

**Box 1:**
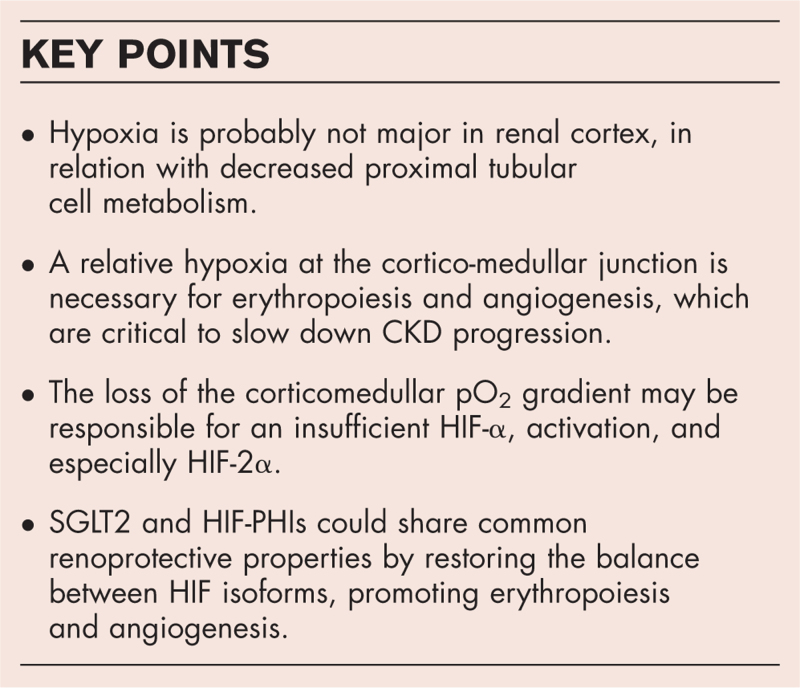
no caption available

## TEXT OF REVIEW

### Hypoxia response

Hypoxia is defined as a state of inadequate oxygen supply to the tissues. Hypoxia consequences on tissue are mainly related to the activation of the inducible factor (HIF) pathway [2]. In conditions where the HIF pathway is activated, such as hypoxia caused by reduced blood flow or oxygen availability, it orchestrates cellular responses to promote adaptation and survival, including angiogenesis, erythropoiesis, and glycolysis [2]. HIFs consist of various oxygen-sensitive α subunits (HIF-1α to -3α) and a stable β subunit (HIF-β), forming heterodimeric complexes. These complexes translocate to the nucleus, where they bind to transcriptional coactivators such as C-AMP Response Element-binding protein (CREB-binding protein)/binding protein p300 (CBP/p300), thereby enhancing the transcription of target genes [3]. HIF-dependent gene regulation orchestrates cellular and tissue adaptation to low oxygen levels. Among the vast array of HIF target genes, numbering potentially over a thousand, are crucial factors like erythropoietin (EPO), transferrin, and vascular endothelial growth factor (VEGF) [2]. Additionally, HIF activation redirects cellular metabolism towards anaerobic pathways, fostering increased glycolytic enzyme expression while inhibiting oxidative metabolism through mechanisms such as upregulating pyruvate dehydrogenase kinase 1 [4,5]. However, prolonged or excessive HIF activation can contribute to pathological processes like inflammation, fibrosis, and tumor progression.

In addition to the effects mediated by the HIF pathway, hypoxia can also exert cellular responses through mechanisms independent of HIF. For instance, hypoxia-induced oxidative stress can activate various signaling pathways, including the mitogen-activated protein kinase (MAPK) and nuclear factor-kappa B (NF-κB) pathways, leading to altered gene expression and cellular responses [6–8]. Moreover, hypoxia-induced reactive oxygen species (ROS) production can directly damage cellular components such as proteins, lipids, and DNA, triggering oxidative stress-mediated pathways such as apoptosis or autophagy [9]. Additionally, hypoxia can modulate cellular metabolism independently of HIF, such as by activating the mammalian target of rapamycin (mTOR) pathway or altering mitochondrial function, leading to metabolic reprogramming to support cell survival under low oxygen conditions [10,11].

HIF-α has two isoforms, HIF-1α and HIF-2α, which exhibit differential tissue expression patterns and regulate distinct sets of target genes. While HIF-1β is universally expressed, HIF-1α is present in all organs, whereas HIF-2α is primarily found in the lungs, heart, and endothelial cells. In the kidneys, HIF-1α is localized within renal tubules, whereas HIF-2α is expressed in endothelial and renal interstitial fibroblast-like (REP) cells [12]. HIF-1α primarily regulates genes involved in glycolytic metabolism, whereas HIF-2α governs the expression of erythropoietin (EPO) and genes crucial for angiogenesis and iron metabolism [13]. Additionally, the role of HIF-3α, though less understood, is speculated to function as a negative regulator of HIF-1α and 2α [14].

The oxygen-dependent regulation of the HIF pathway involves the enzymatic activity of prolyl-4-hydroxylase domain (PHD1–3) enzymes and the asparagine hydroxylase factor inhibiting HIF (FIH) [12]. PHDs catalyze the hydroxylation of HIF-α isoforms, leading to their degradation via ubiquitination mediated by the von Hippel Lindau (VHL) protein, whereas FIH-mediated asparagine hydroxylation prevents HIF heterodimer interaction with CBP/p300, thereby reducing HIF transactivation activity for specific target genes [15].

### Are the kidneys hypoxic during chronic kidney disease?

Next to the heart, the kidney exhibits the second-highest metabolic rate (>400 kcal/kg tissue/day), utilizing approximately 7% of the body's total daily energy despite its comparatively low weight [16]. This substantial energy demand is attributed to the transport and active reabsorption of nutrients and electrolytes within the tubules, as well as the active secretion of unnecessary compounds [17]. The proximal tubule is the most metabolically active segment of the nephron and has the highest oxygen-consuming rate in the kidney. During CKD, proximal tubule metabolism and therefore oxygen consumption decreases.

Several works have demonstrated arguments for a relative hypoxia during kidney disease. Blood Oxygenation Level Dependent-Magnetic Resonance Imaging (BOLD-MRI), for example, showed a correlation between low cortical oxygenation and CKD stage [18–20]. In diabetic nephropathy models, hypoxia was also found, even at early stages [21,22], which was also the case in models of hypertensive nephropathy [23].

However, the assessment of hypoxia in CKD is hindered by limited resolution of available methods for understanding spatial and time-dependent differences in kidney tissue oxygenation. Techniques such as pimonidazole staining, microelectrode-dependent measurements, analysis of HIF-α stabilization/transcriptional activity, and bioimaging methods like BOLD-MRI offer insights but suffer from limitations [24]. While some techniques provide spatial detection or quantitative oxygen detection, they are often indirect or biased, failing to reflect intra-tissue oxygen levels accurately [25].

More recent data can raise some questions regarding hypoxia during CKD. Clinical studies using BOLD-MRI hint at a potential predictive role of low cortical oxygenation in renal function decline, yet direct correlations with CKD stage remain elusive [26]. A recent study in adenine nephropathy using micro-electrodes did not observe a large hypoxia during the disease [27]. Finally, we observed only late and patchy hypoxia in a model of progressive proteinuric kidney disease, with only some remnant tubuli being hypoxic [28].

Even if hypoxia is present during different types of renal disease, its deleterious role is not completely clear. Most of the deleterious role of hypoxia have been attributed to HIF activation. This was mostly based on genetic studies showing a detrimental role of uncontrolled and constant HIF activation.

More recent work tends to show that the HIF pathway may not be activated as strongly as initially thought during CKD. In diabetes, several studies have shown that there is a downregulation of the HIF pathway, potentially related to hyperglycemia [29]. In our recent study, the HIF pathway was not activated in a progressive model of proteinuric kidney disease, in urinary tract obstruction, or in a late post ischemic model of kidney disease [28]. This was also the case in a chronic adenine model [27,30^▪▪^]. Finally, in a recent study about the identification of druggable pathways leading to failed repair, hypoxia sensing was not on the top list, although this may not exclude its participation [31^▪▪^]. Finally, the fact that EPO production decreases during CKD and is still stimulable by PHD inhibitor questions a large activation of the HIF pathway, at least of HIF2.

These observations are however limited by the fact that assessing the activation of the HIF pathway is not an easy task and suffers from several limitations as discussed below.

Altogether, although the presence of hypoxia during CKD has been largely studies, doubts still occur due to the methods and models used and on its role as a factor contributing to renal disease progression. More powerful tools may answer these specific questions.

### Is hypoxia detrimental during chronic kidney disease?

The kidneys serve as sophisticated oxygen sensors, playing a crucial role in maintaining tissue oxygenation and homeostasis. The kidneys use only 10% of the oxygen delivered by the blood flow [32], and most of the oxygen goes to the renal cortex. The renal medulla is physiologically hypoxic, with oxygen partial pressure as low as 10 mmHg [33]. This gradient between cortex and medulla, called the corticomedullary gradient, allows a subtle sensing of the pO2 by the renal erythropoietin (EPO) producing cells [34], located at the corticomedullary junction. The maintenance of this gradient is crucial for efficient EPO production; therefore, there is a relative need for the presence of hypoxia in the medulla.

Apart from the doubts about hypoxia's presence in late-stage renal disease, we can therefore question its detrimental role in CKD, especially considering conflicting findings such as improved disease course in some experimental models exposed to hypoxia [21,35]. In recent studies in different animal models, exposition to external hypoxia did reveal nephroprotective [30^▪▪^].

The role of HIF activation itself is a matter of debate; since the regulation of HIF-α does not primarily occur at the transcriptional level, mRNA analyses of HIF-α typically fail to provide a clear indication of HIF pathway activation. While examining mRNA levels of HIF target genes can offer some insights, this approach may be influenced by other gene expression regulations, complicating interpretation. Furthermore, protein analyses of HIF pathway components or downstream targets are restricted by the quality of available antibodies. Moreover, due to the rapid regulation of HIF-α protein, animal euthanasia and tissue collection procedures can introduce bias by altering tissue pO_2_ levels [36].

In addition to the difficulties in estimating the degree of HIF activation, there is also a difference between the type of cells where the pathway is activated. Indeed, a systemic inactivation of both HIF-1α and HIF-2α worsens renal outcome in the unilateral urinary tract obstruction (UUO) model of CKD [37], although a tubular HIF-1α knock-out was protective in another study [38]. HIF-1α activation in proximal tubular cells was protective in diabetic nephropathy[39], and its deletion detrimental [40,41]. The timing of the activation seems important; tubular-specific knockout of HIF-2α exacerbated early histological lesions in the adenine model but protected against fibrosis and renal function decline in later stages [42^▪^].

Finally, the difference between the two isoforms seems important. HIF-1α is more dedicated to glycolysis, whose overactivation in tubular cells seem detrimental in CKD, whereas HIF-2α regulated EPO production and angiogenesis, crucial for renoprotection [43,44]. Some authors describe mutual antagonist actions of HIF-1α and HIF-2α, with more pro-fibrotic actions of HIF-1α and antifibrotic properties of HIF-2α. Also, HIF-2α decreases oxidative stress, transactivating antioxidative enzymes. In diabetic nephropathy, HIF-2α seems to be insufficiently activated, and this could explain why angiogenesis and erythropoiesis are impaired [45].

### Stimulation of hypoxia-inducible factor: the case of prolyl-4-hydroxylase domain inhibitors in fibrosis progression

Recently, development in HIF-PHIs have shown promising results in renal anemia treatment. Roxadustat, vadadustat, and daprodustat are among the HIF stabilizers that have shown promise in clinical trials. These small molecule drugs mimic the physiological response to hypoxia by stabilizing HIF, which subsequently upregulates EPO production and enhances erythropoiesis. Unlike traditional erythropoiesis-stimulating agents (ESAs), which directly target EPO receptors, HIF stabilizers induce endogenous EPO production in a more physiological manner. This mechanism offers several potential advantages, including improved responsiveness to fluctuations in oxygen levels and reduced risk of EPO resistance. Clinical studies have demonstrated the efficacy and safety of HIF stabilizers in increasing hemoglobin levels and reducing the need for transfusions in patients with CKD-associated anemia [46,47]. The efficiency of this class of drugs is confirmed for hemoglobin level maintenance, but they also might have an impact on CKD progression. In preclinical models, HIF-PHIs have shown renoprotective effects in a mouse model of obesity [48], in a chronic model of tubulo-interstitial nephritis [49], in the rat subtotal nephrectomy model [50]. Some effects towards ROS production and glycogen retaining have been described in ischemic acute kidney injury models [51], and globally antifibrotic properties in these models [52] and in cisplatin AKI models [53]. This description contrast with genetic data and reflect maybe the difference between transient and constant activations of the pathways, or of a more pro-eminent activation of HIF-2α compared to HIF-1α [54]. Despite these promising preclinical results, the results in clinical studies are so far unconclusive regarding progression of renal disease.

Other potential targets could include FIH inhibitors, besides PHD inhibitors. FIH is relatively neglected in the study of HIF pathway regulation, but a study published by our group showed that it was upregulated in CKD, potentially contributing to the insufficient HIF pathway activation. FIH inhibitors were tested in CKD models *in vivo* and showed promising results on fibrosis deposition and a trend towards limiting GFR decrease [28].

### Hypoxia-inducible factor stimulation: the case of SGLT2 inhibitors

The beneficial effects of SGLT2 inhibitors in CKD is now clearly demonstrated, but the precise mechanisms leading to this level of nephroprotection remain uncertain. A decrease in glomerular hyperfiltration is often cited as a mechanism, but it is probably not the only factor. The effect of SGLT2 inhibitors on HIF pathway is debated, as some preclinical studies show a decrease in HIF-1α activity [55,56], and some others an activation of the pathway [57^▪^]. However, the reduction of tubular workload in S1 segment by decreasing glucose reabsorption should lead to a decreased oxygen consumption by the same mechanism. As a consequence, it is likely to increase tubular workload in downstream segments, leading to increased oxygen needs and therefore reducing oxygen partial pressure. This will in turn lead to a stimulation of the HIF pathway and could explain the effect of SGLT2 inhibitors on erythropoiesis and angiogenesis. Indeed, SGLT2 inhibitors decreased capillary rarefaction in a model of ischemia-reperfusion injury through VEGF stimulation [58]. This could also be related to a preferential effect on HIF-2α stimulation that could be nephroprotective [59,60].

## CONCLUSION

This review illuminates the complex interplay between CKD, hypoxia, the HIF pathway, and the therapeutic potential of SGLT2 inhibitors and HIF prolyl hydroxylase inhibitors (HIF-PHIs). Firstly, we hypothesize that hypoxia is not as important as hypothesized in the renal cortex, in relation with decreased proximal tubular cell metabolism. This may lead to the loss of the corticomedullar pO_2_ gradient, which may be in turn responsible for an insufficient HIF-α activation, and especially HIF-2α. Other factors such as inhibitors or inflammation may also explain the inhibition of hypoxia sensing pathways observed in several settings.

The deleterious effect of HIF activation during CKD is also debated, with most observations coming from genetic stable overexpression of the pathway, whereas physiological activation of HIF may be protective.

**FIGURE 1:**
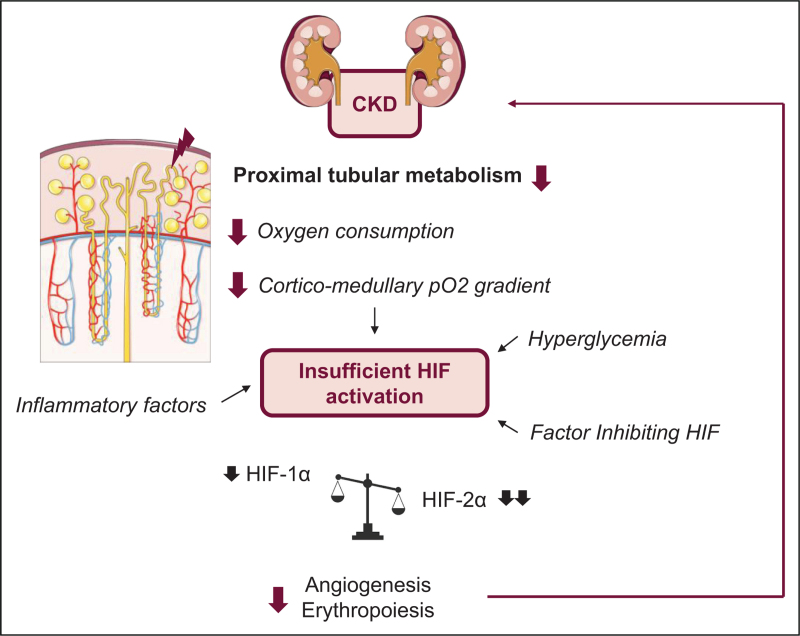
Proposed mechanism for insufficient HIF activation.

Finally, the potential use of novel drugs such as PHD inhibitors and SGLT2 inhibitors, stabilizing HIF and inducing EPO production also question the dogma of the detrimental effect of this pathway during renal disease (Fig. 1).

## Acknowledgements

*Anna Faivre drafted the review. Sophie de Seigneux provided intellectual input and corrected the draft.*

### Financial support and sponsorship

*Anna Faivre received a Swiss National Fund (SNF) grant for her thesis and a research grant by the de Reuter foundation. Sophie de Seigneux is a recipient of a SNF grant. There is no direct source of funding for this review.*

### Conflicts of interest

*Anna Faivre received consultancy fees from Astellas. Sophie de Seigneux received consultancy fees from Astellas, AstraZeneca, Bayer, and Vifor Pharma.*

## REFERENCES AND RECOMMENDED READING

Papers of particular interest, published within the annual period of review, have been highlighted as:

▪ of special interest
▪▪ of outstanding interest
